# Prediction of age at onset in Parkinson’s disease using objective specific neuroimaging genetics based on a sparse canonical correlation analysis

**DOI:** 10.1038/s41598-020-68301-x

**Published:** 2020-07-15

**Authors:** Ji Hye Won, Mansu Kim, Jinyoung Youn, Hyunjin Park

**Affiliations:** 1 Department of Electronic and Computer Engineering, Sungkyunkwan University, Suwon, Korea; 2 Center for Neuroscience Imaging Research, Institute for Basic Science, Suwon, Korea; 3 Department of Neurology, Samsung Medical Center, Sungkyunkwan University School of Medicine, Irwon-ro 81, Gangnam-gu, Seoul, 06351 Korea; 4 Neuroscience Center, Samsung Medical Center, Seoul, Korea; 5 School of Electronic and Electrical Engineering, Sungkyunkwan University, Suwon, 16419 Korea

**Keywords:** Computational neuroscience, Parkinson's disease

## Abstract

The age at onset (AAO) is an important determinant in Parkinson’s disease (PD). Neuroimaging genetics is suitable for studying AAO in PD as it jointly analyzes imaging and genetics. We aimed to identify features associated with AAO in PD by applying the objective-specific neuroimaging genetics approach and constructing an AAO prediction model. Our objective-specific neuroimaging genetics extended the sparse canonical correlation analysis by an additional data type related to the target task to investigate possible associations of the imaging–genetic, genetic–target, and imaging–target pairs simultaneously. The identified imaging, genetic, and combined features were used to construct analytical models to predict the AAO in a nested five-fold cross-validation. We compared our approach with those from two feature selection approaches where only associations of imaging–target and genetic–target were explored. Using only imaging features, AAO prediction was accurate in all methods. Using only genetic features, the results from other methods were worse or unstable compared to our model. Using both imaging and genetic features, our proposed model predicted the AAO well (r = 0.5486). Our findings could have significant impacts on the characterization of prodromal PD and contribute to diagnosing PD early because genetic features could be measured accurately from birth.

## Introduction

Parkinson’s disease (PD) is the second most common neurodegenerative disorder and presents with various motor and non-motor symptoms^1^. Like other neurodegenerative diseases, age at onset (AAO) in PD patients is important because it is related to clinical characteristics and progression^2–7^. Young-onset PD patients show a good response to levodopa, more dyskinesia, dystonia, and slower progression^2–4^, while late-onset PD patients demonstrate more axial symptoms, cognitive impairment, and genitourinary symptoms^5–7^. As such, there are differences in PD characteristics according to AAO. Many previous studies revealed that neuroimaging and genetic factors play an important role for AAO in PD patients^8–12^. Finding the significant features from both neuroimaging and genetic factors related to AAO in PD could enable understanding of the pathological mechanisms underlying the associations between AAO and PD.

Neuroimaging genetics is an emergent transdisciplinary approach, where the associations between genetic variations and neuroimaging measures are explored. Neuroimaging genetics is more sensitive than a conventional genome-wide association study (GWAS) as it integrates richer neuroimaging information compared to binary diagnosis. Neuroimaging provides a rich quantitative characterization of disease and promises to aid in identifying genetic variations that are correlated with the clinical variables of interest. Neuroimaging genetics is different from conventional GWAS where associations between genetic factors and phenotypes are directly explored. By identifying the associations between genetic factors and imaging measurements, neuroimaging genetics intends to model and understand how genetic factors influence the structure or function of the human brain. Canonical correlation analysis (CCA) is a popular method of imaging genetics to combine high-dimensional data types. The CCA finds the best linear transformations for imaging and genetic features so that the correlation between imaging and genetic can be maximized. In practice, the sparse CCA (SCCA) method is adopted to find a sparse (i.e., only a few components) set of features through regularization, such as with least absolute shrinkage and selection operator (LASSO). The existing SCCA was focused only on finding the best linear transformations for imaging and genetic features, respectively. Therefore, the resulting set of features has a high imaging–genetic association, but it does not necessarily have a strong association with the disease being studied. This makes interpreting the selected features challenging as we are not sure of the feature–target disease association. To improve the interpretation, we extended the sparse CCA to include a third data type related to the target task (i.e., AAO of PD), which leads to simultaneously exploring the associations of the imaging–genetic, genetic–target, and imaging–target pairs. Our rationale was to explore all three possible pair-wise associations to leverage the available data fully. We named the approach objective specific neuroimaging genetics emphasizing the third added data type related to the main objective.

In this study, we applied the objective-specific neuroimaging genetics approach to identify genetic and imaging features associated with AAO in PD patients, not in healthy controls. We also aimed to construct an analytical model to predict the AAO of PD using the identified genetic and imaging features simultaneously. Neuroimaging and single nucleotide polymorphisms (SNPs) data from the Parkinson’s Progression Markers Initiative (PPMI) were obtained. Neuroimaging features that can reflect the differences in the brain structure and function with respect to AAO of PD were computed from the fractional anisotropy (FA) of diffusion tensor imaging (DTI) that is a sensitive method to assess PD pathophysiology and severity^13^. Then, we applied our proposed method with the FA values, PD-related SNP alleles, and AAO as three input data types. The results were then compared with existing methods.

We hope to better understand how genetic variations of specific risk genes lead to alterations in the brain structure and function with neuroimaging genetics. Such an understanding could have tremendous potential for accurately diagnosing and improving therapy for PD. The imaging and genetic feature used to predict AAO of PD could help with characterizing the prodromal period of PD. This study focused on AAO in PD patients only still, our study could be extended to predict diagnosis by replacing the AAO term with diagnosis status and ultimately contribute to the early diagnosis of PD. In particular, our study could make a significant contribution to preventive medicine in PD, combining the identified SNPs with the known environmental factors of PD. The neurodegenerative process has already substantially progressed when the diagnosis of PD is established based on the widely accepted clinical criteria^14^. However, our identified SNPs might serve as early biomarkers for AAO in PD as they can be measured accurately from birth.

## Methods

### Subjects

This study was a retrospective analysis, and institutional review board (IRB) approval was obtained from Sungkyunkwan University. Our study was performed in full accordance with the local IRB guidelines. Informed consent was obtained from all subjects. Our study data of 146 PD patients were obtained from the PPMI database^15^. We used DTI, T1-weighted MRI, and DNA genotyping data. Inclusion criteria were aged 30 years or older, diagnosis of PD (based on one of the following: presence of 1 asymmetrical resting tremor or 2 asymmetrical bradykinesia or 3 at least 2 of resting tremor, bradykinesia, and rigidity), disease duration of 1–24 months, Hoehn and Yahr (H&Y) stage of 1–2, and presence of the T1-MRI, DTI, and genotyping data. AAO was based on patients’ recollection of the first parkinsonian motor symptom^3^. Additionally, we divided the patients into four subgroups based on AAO (younger than 50 years, 50–59 years, 60–69 years, and 70 years or older), according to Pagano et al^3^. The demographic and clinical data of enrolled subjects were illustrated in Table 1.

**Table 1:** Demographic and clinical data of the enrolled subjects.

	All n = 146	Age at onset, years				*p* value
		Years $<$ 50 (group1) n = 24	50 $\le$ years $<$ 60 (group2) n = 35	60 $\le$ years $<$ 70 (group3) n = 54	years $\ge$ 70 (group4) n = 33	
Age at onset of PD	61.35 $\pm$ 9.73	46.22 $\pm$ 3.92	54.82 $\pm$ 2.90	65.03 $\pm$ 2.97	73.23 $\pm$ 2.75	–
Sex, male, % (n)	65.07 (95)	70.83 (17)	45.71 (16)	79.63 (43)	57.58 (19)	–
PD duration, month	7.55 $\pm$ 6.72	6.25 $\pm$ 6.75	7.09 $\pm$ 5.87	8.30 $\pm$ 0.98	7.79 $\pm$ 7.22	0.63
H&Y stage	1.68 $\pm$ 0.54	1.71 $\pm$ 0.46	1.51 $\pm$ 0.56	1.67 $\pm$ 0.55	1.88 $\pm$ 0.48*	0.04
MDS-UPDRS total	32.32 $\pm$ 14.97	30.00 $\pm$ 15.90	32.11 $\pm$ 12.19	30.59 $\pm$ 14.99	37.06 $\pm$ 16.47	0.20
UPDRS Part1	5.31 $\pm$ 4.30	4.71 $\pm$ 4.97	6.00 $\pm$ 4.78	5.04 $\pm$ 4.26	5.45 $\pm$ 3.27	0.66
UPDRS Part2	5.41 $\pm$ 3.96	5.88 $\pm$ 4.97	5.06 $\pm$ 3.82	5.11 $\pm$ 3.60	5.94 $\pm$ 3.97	0.69
UPDRS Part3	21.45 $\pm$ 10.20	19.21 $\pm$ 8.28	$20.83\pm$ 9.11	20.43 $\pm$ 9.94	25.42 $\pm$ 12.16	0.08
UPDRS Part4	0.15 $\pm$ 0.72	0.21 $\pm$ 0.72	0.23 $\pm$ 0.84	0.02 $\pm$ 0.14	0.24 $\pm$ 1.06	0.40

*H&Y* Hoehn and Yahr, *MDS-UPDRS* Movement Disorder Society-Unified Parkinson’s Disease Rating Scale.

*p* value computed from an ANOVA of the four groups.

*p value < 0.05 for comparing group 3 and 4.

### Neuroimaging and genotype data

MRI data were obtained using a standard protocol for 3 T scanners. T1-weighted MRI were acquired using the following imaging parameters (repetition time [TR] = 2,300 ms, echo time [TE] = 2.98 ms, image matrix = 240 × 256 × 176, and voxel resolution = 1 × 1 × 1 mm^3^). In the case of DTI, the acquisition protocol included a 3D magnetization prepared rapid gradient-echo sequence and a 2D single-shot echo-planar DTI sequence (TR/ TE = 5,900/88 ms, 2 mm^3^ isotropic resolution; 72 contiguous slices, twofold acceleration, axial–oblique aligned along the anterior–posterior commissure, diffusion-weighting along 64 gradient directions with a b value of 1,000 s/mm^2^). Further details of the MRI acquisition and processing are available from the PPMI website (https://www.ppmi-info.org/). Most DTI scans were obtained with cardiac gating except for a few cases (less than 5%) in which a clear cardiac signal was not available.

All genetic data (i.e., SNP) were genotyped by NeuroX genotyping arrays. We compiled the quality-controlled data into a single VCF file to extract the variants specified (i.e., gene, rsID, etc.) and splatted multi-allelic sites into two using BCFtools^16^. We performed the preprocessing of SNP data according to the protocol of the ENIGMA^17^. Minor allele frequency (< 0.01), genotype call rate (< 95%), Hardy–Weinberg equilibrium *p* value (< 10^–6^), and sex-matching were performed in PLINK v1.9 software^18^. Then, we converted this VCF file into the binary PLINK format to enforce a minimum genotyping quality (GQ) score threshold of 20 and recode the data into a text file containing the number of copies of the minor allele of each variant for each subject using the PLINK^18^.

**Figure 1:**
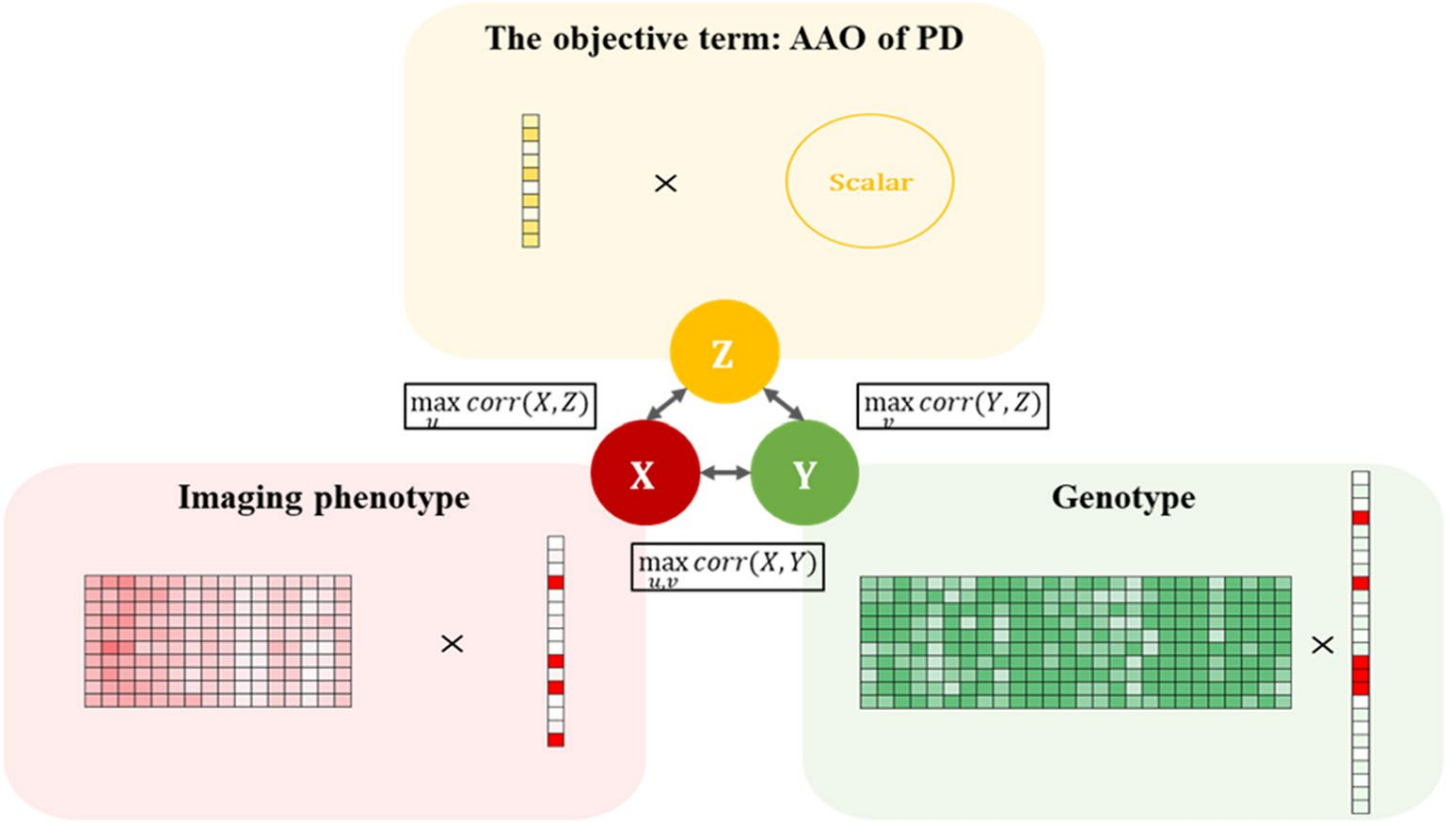
Schematic of our objective specific sparse canonical correlation analysis (os-SCCA) model. Our os-SCCA model is an extension of the sparse canonical correlation analysis. It includes an objective term (i.e., AAO of PD) to investigate the possible associations of the imaging–genetic, genetic–target, and imaging–target pairs simultaneously.

We mainly used the two types of high-dimensional information (i.e., DTI and genetic data) for our study. We extended the SCCA to incorporate the third data type related to the target task (AAO of PD). This led to exploring imaging-genetic associations as well as target-imaging and target-genetic associations simultaneously (Fig. 1). Details regarding the procedures are provided later in this study.

### DTI processing

Before DTI processing, T1-weighted images need to be processed first. The T1-weighted images were skull-stripped and nonlinearly registered onto the standard Montreal Neurological Institute (MNI) spatial frame using the FMRIB Analysis Group Software Library (FSL, https://fsl.fmrib.ox.ac.uk/fsl/fdt)^19^. The DTI data were processed using the diffusion tensor analysis toolkit (FDT) of FSL. Head motion and image distortions induced by eddy currents were corrected by applying a 3D full-affine alignment of each image to the mean no-diffusion-weighting (b0) image. DTI data were averaged and concatenated after the correction of distortion. We generated voxel-wise maps of FA, which quantifies how elongated the diffusion tensor ellipsoid is. Additionally, it is known to be sensitive to a wide range of pathologies^20^. FA maps were registered onto the common MNI space using the same procedure to process T1 MRI. We adopted the automated anatomical labeling (AAL) atlas defined on the MNI space to specify the region of interests (ROIs). We finally calculated the FA values of 90 ROIs using the sample mean of voxel-wise FA values from each ROI.

### Objective-specific imaging genetic associations

Below we use boldface lowercase letters to denote a vector and boldface uppercase letters to denote a matrix. Given datasets ${\varvec{X}}\in {\mathbb{R}}^{n\times p}, {\varvec{Y}}\in {\mathbb{R}}^{n\times q},$ and ${\varvec{Z}}\in {\mathbb{R}}^{n\times 1},$ with *n* samples, where ***X*** denotes *p* features of neuroimaging (i.e., FA values) and ***Y*** denotes *q* features of genetics (i.e., the count of the non-reference allele (0, 1, 2) for each SNP), and ***Z*** denotes features related to target objective (i.e., AAO of patients with PD). Our proposed objective-specific SCCA (os-SCCA) is a special case of conventional three-way SCCA (TSCCA), where the third data type is from the target task. This leads to the optimization of three pairwise SCCAs simultaneously^21,22^. By replacing one data type of TSCCA with objective-relevant information, we can find imaging genetic associations that are relevant to a particular task (i.e., AAO of PD). The formulation is defined as follows:

1 $$\begin{gathered} \mathop {\max }\limits_{{\boldsymbol{u},\boldsymbol {~v}}} \boldsymbol {u}^{{\text{T}}} \boldsymbol {X}^{{\text{T}}} \boldsymbol {Yv} + w\boldsymbol {v}^{{\text{T}}} \boldsymbol {Y}^{{\text{T}}} \boldsymbol {Z} + w\boldsymbol {Z}^{{\text{T}}} \boldsymbol {Xu,} \hfill \\ s.t.~~\boldsymbol {u}^{{\text{T}}} \boldsymbol {X}^{{\text{T}}} \boldsymbol {Yv} \le 1,\boldsymbol {v}^{{\text{T}}} \boldsymbol {Y}^{{\text{T}}} \boldsymbol {Z} \le 1,\boldsymbol {Z}^{{\text{T}}} \boldsymbol {Xu} \le 1,{\text{~}}\boldsymbol {u}_{1} \le {\text{c}}_{1} ,~\boldsymbol {v}_{1} \le {\text{c}}_{2} \hfill \\ \end{gathered}$$

where ${\varvec{u}}$ and ${\varvec{v}}$ were the corresponding canonical loading vectors, and $w$ was scalar. Our approach is a simple version of CCA where only the first principal directions were considered to maximize the canonical correlation value. The canonical loading vector u for X (n × p matrix of neuroimaging data) contains information regarding what regions to choose over p regions. In the same vein, you can think of $w$ as canonical loading vector for Z (i.e., AAO values). Since ***Z*** is an n × 1 vector *w* become a scalar (1 × 1 vector). We can rewrite the objective functions for os-SCCA as follows^21^:

2 $$\underset{{\varvec{u}},\boldsymbol{ v}}{\mathrm{min}}-{{\varvec{u}}}^{\mathrm{T}}{{\varvec{X}}}^{\mathrm{T}}{\varvec{Y}}{\varvec{v}}-{w{\varvec{v}}}^{\mathrm{T}}{{\varvec{Y}}}^{\mathrm{T}}{\varvec{Z}}-w{{\varvec{Z}}}^{\mathrm{T}}{\varvec{X}}{\varvec{u}}+{\lambda }_{u}{\Vert {\varvec{u}}\Vert }_{1}+{\lambda }_{v}{\Vert {\varvec{v}}\Vert }_{1}$$

where ${\lambda }_{u}$ and ${\lambda }_{v}$ were regularization parameters. The $l_1$ penalty, controlled by ${\lambda }_{u}$ and ${\lambda }_{v}$, was applied to induce sparsity and prevent overfitting^21^.

### Imaging–genetic feature selection from os-SCCA and construction of prediction models

Due to a limited number of samples, we adopted a nested five-fold cross-validation. For the outer loop, we separated the data into training and test sets using a fivefold split. For the inner loop, the training data were subject to another fivefold cross-validation where the inner training set was used to train the model and the remaining set was used as the validation set to tune the hyperparameters of the model. The value of w was set using the formula given in a previous study adjusted for the dimensions of ***Z***^21^. Optimal value of regularization parameters (i.e., ${\lambda }_{u}$, ${\lambda }_{v}$) was determined within each training set via internal fivefold cross-validation (CV).

3 $$\mathrm{CV} = \frac{1}{5}\sum_{i=1}^{5}\frac{1}{3}\left(corr({{\varvec{X}}}_{{\varvec{i}}}{{\varvec{u}}}_{-{\varvec{i}}},\boldsymbol{ }{{\varvec{Y}}}_{{\varvec{i}}}{{\varvec{v}}}_{-{\varvec{i}}})+corr({{\varvec{X}}}_{{\varvec{i}}}{{\varvec{u}}}_{-{\varvec{i}}},\boldsymbol{ }{{\varvec{Z}}}_{{\varvec{i}}}w)+corr({{\varvec{Y}}}_{{\varvec{i}}}{{\varvec{v}}}_{-{\varvec{i}}}{{\varvec{Z}}}_{{\varvec{i}}}w)\right)$$

where ${{\varvec{X}}}_{{\varvec{i}}}$,.${{\varvec{Y}}}_{{\varvec{i}}}$, and ${{\varvec{Z}}}_{{\varvec{i}}}$ denoted the *i*-th subset of the validation set and ***u***_*-I*_ and ***v***_*-i*_, denoted the estimated loading vectors from the datasets except for the *i*-th subset (training set, ***X***_-*i*_, ***Y***_-*i*_, and ***Z***_-*i*_) in the inner loop. We calculated the average metric score over the five folds in (3) and chose the average as the hyperparameters. Coming back to the outer loop, we used data from four folds to train the regression model and applied the learned model to the left-out test fold. We repeated this process five times with a different fold left-out each time. The software code used in this study is available at code-sharing website (https://github.com/Ji-Hye-Won/os-tscca).

We applied FA values, the number of minor alleles (i.e., 0, 1 or 2) of PD-related SNP, and AAO of PD as three inputs for training samples to os-SCCA. The elements with non-zero coefficients of the loading vectors were the selected imaging and genetics features. Then, we constructed random forest regression models based on the selected imaging and genetic features to predict the AAO of PD. We trained a random forest of 500 regression trees using the selected features. Three regression models with only the selected imaging features, genetic features, and combined imaging and genetic features were built. This resulted in five sets of performance metrics of the models, which were averaged. We assessed the performance of the prediction using Pearson’s correlation between the actual and predicted AAO of PD in the test fold. The root mean square error (RMSE) was also used^23^.

### Over-representation enrichment analysis

The selected SNPs were annotated with the genes, and they were processed with an over-representation enrichment analysis (ORA) using an online database WebGestalt (https://www.webgestalt.org/2019/). The ORA compares sets of genes annotated to pathways to a list of the identified genes that are significantly deferentially expressed. A hypergenometric test was adopted to detect an over-representation of the high-ranking genes among all the genes in a given category. The whole genome was selected as the reference gene list.

### Imaging-genetic feature selection from other methods

The selected features and prediction models from the os-SCCA were compared with existing sparse multi-view feature selection methods. We compared our approach with LASSO and the minimum redundancy maximum relevance (mRMR), where only associations of genetic-target and imaging-target were explored. Our method extracts significant SNP and imaging features at the same time. Contrastingly, LASSO and mRMR select significant features of a target variable (i.e., AAO of PD) directly from larger sets of candidate predictors corresponding to features from imaging and genetic separately. In the case of mRMR, the number of selected features is a user parameter. This number was set as the same number of features obtained from our os-SCCA. We also constructed random forest regression models using imaging, genetic, and combined features from LASSO and mRMR based the training set data. We validated the models by applying them to test sets. In summary, the same procedures were applied using different selected features from the LASSO and mRMR approaches.

## Results

### Selected imaging and genetic features from the os-SCCA approach

We used FA values and genetic data of 146 PD patients from the PPMI database^15^. The FA values of 90 regions were computed from the DTI preprocessing. The 72 allelic statuses were selected from Parkinson’s disease-associated variants for PPMI subjects. Our analysis is different from GWAS as we performed our experiments with only SNPs related to PD. Thus, the identified SNPs were not subject to statistical rigors of GWAS and should be interpreted as important variables of the prediction model. We applied the os-SCCA approach using these two types of data and target information (i.e., AAO of PD) in a five-fold cross-validation. The identified features were similar in the five sets of results. We only reported results that were common to all five sets.

We identified FA of 14 ROIs related to both SNPs and AAO of PD in all five training sets. The 14 selected regions were as follows: left and right precentral gyrus (PreCG); left and right median cingulate and paracingulate gyri (DCG); left and right posterior cingulate gyrus (PCG); left and right superior occipital gyrus (SOG); left and right lenticular nucleus, putamen (PUT); left and right lenticular nucleus, pallidum (PAL); and left and right thalamus (THA).

We identified 24 variants related to both the FA and AAO of PD. The 24 selected variants were as follows: rs823118, rs4653767, rs6430538, rs353116, rs12497850, rs11724635, rs6812193, rs3910105, rs199347, rs591323, rs13294100, rs11060180, rs8005172, rs14235, rs737866, rs174674, rs740603, rs165656, rs6269, rs4633, rs2239393, rs4818, rs4680, and rs165599. Table 2 provides a summary of detailed information on the 24 variants. The identified SNPs were annotated with the genes, and we found that most of the resulting variants were on the COMT gene on chromosome 22.

**Table 2:** Selected SNPs from os-SCCA.

CHR^a^	RSID for the SNP	BP^b^	MA^c^	Associated gene
1	rs823118	205754444	C	NUCKS1
1	rs4653767	226728377	C	ITPKB
2	rs6430538	134782397	C	ACMSD/TMEM163
2	rs353116	165277122	T	SCN3A/ SCN2A
3	rs12497850	48711556	G	NCKIPSD/CDC71/IP6K2
4	rs11724635	15735478	C	BST1
4	rs6812193	76277833	T	FAM47E/STBD1
4	rs3910105	89761420	G	SNCA
7	rs199347	23254127	G	GPNMP
8	rs591323	16839582	A	MICU3/FGF20
9	rs13294100	17579692	T	SH3GL2
12	rs11060180	122819039	G	OGFOD2/CCDC62
14	rs8005172	88006268	T	GALC/GPR65
16	rs14235	31110472	A	ZNF646/KAT8/BCKDK
22	rs737866	19942586	C	COMT
22	rs174674	19946502	A	COMT
22	rs740603	19957654	A	COMT
22	rs165656	19961340	G	COMT
22	rs6269	19962429	G	COMT
22	rs4633	19962712	C	COMT
22	rs2239393	19962905	G	COMT
22	rs4818	19963684	G	COMT
22	rs4680	19963748	A	COMT
22	rs165599	19969258	G	COMT

^a^*CHR* chromosome number.

^b^*BP* base-pair location in hg38 coordinates.

^c^*MA* minor allele of variant based on PPMI sample.

### Over-representation enrichment analysis results

**Figure 2:**
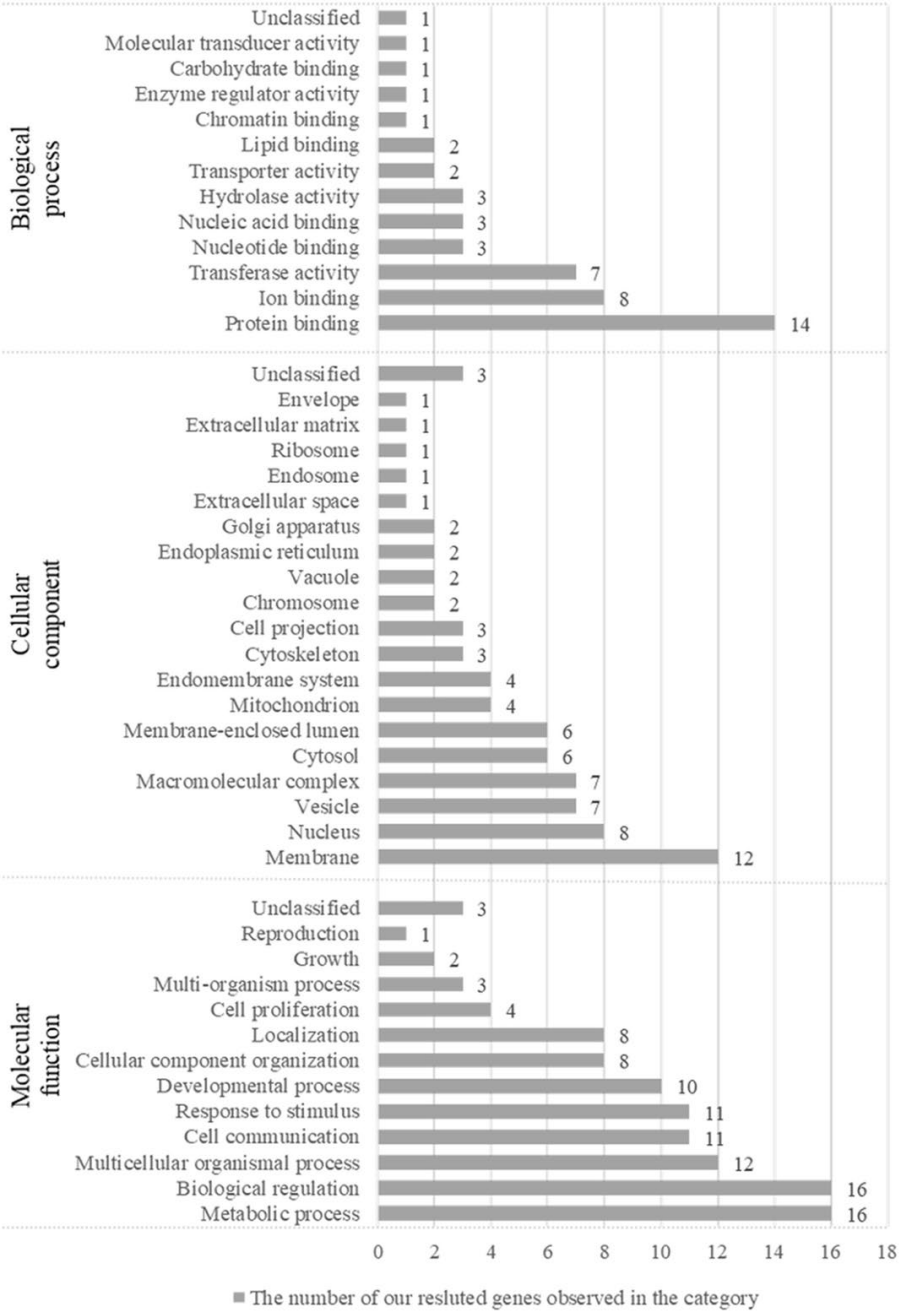
Summary of the over-representation enrichment analysis from the identified SNPs. The plot shows the significant gene ontology analysis results from the 25 genes of the 24 identified SNPs. Genes were annotated with the three selected functional categories (corresponding to the categories written vertically), which were also in the reference list. The length of the bar represents the number of the identified genes observed in the reference gene list.

Table 2 provides a summary of the identified 24 SNPs, including their corresponding genes. After annotating these SNPs, genes were analyzed using an ORA. The list contained 25 gene names in which 23 genes are mapped to 23 unique entrezgene IDs. Among 23 unique entrezgene IDs, 20 genes were annotated to the selected functional categories and the reference list, which were used for the enrichment analysis. A significant gene ontology analysis included negative regulation of the cellular amine metabolic process (false discovery rate [FDR] = 0.0324) and regulation of the cellular amine metabolic process (FDR = 0.0199). The top 10 categories were identified as enriched categories. These are shown in Fig. 2.

### Selected imaging and genetic features from mRMR

The same number of features were used between our approaches and mRMR to enable a fair comparison. Each feature was ranked using the feature selection (FS) score provided from the mRMR, and we analyzed only a few features (i.e., the same number from os-SCCA) with the highest FS scores. The FS scores were typically high, above 0.2, for the first few imaging features and then dropped significantly to 10^−16^ afterward. The imaging features of the five folds constantly above the threshold of 0.2 in a descending order based on the FS score were as follows: left PCG, left lenticular nucleus, PAL, right PAL, right PCG. The FS scores of the genetic features decreased gradually without sudden drops. The 26 genetic variants of the five folds are shown in Table 3, including rs3910105, rs823118, rs11060180, rs8005172, rs34043159, and rs6812193.

**Table 3:** Selected SNPs from mRMR.

CHR	RSID for the SNP	BP	MA	Associated gene
1	rs823118	205754444	C	NUCKS1
2	rs34043159	101796654	C	IL1R2/MAP4K4
2	rs6430538	134782397	C	ACMSD/TMEM163
2	rs353116	165277122	T	SCN3A/SCN2A
3	rs4073221	18235996	G	SATB1
3	rs12497850	48711556	G	NCKIPSD/CDC71/IP6K2
4	rs34311866	958159	C	TMEM175
4	rs11724635	15735478	C	BST1
4	rs6812193	76277833	T	FAM47E/STBD1
4	rs356181	89704988	G	SNCA
4	rs3910105	89761420	G	SNCA
4	rs4444903	109912954	G	EGF
8	rs2280104	22668467	T	BIN3
10	rs10906923	15527599	C	FAM171A1/ITGA8
11	rs329648	133895472	T	MIR4697
12	rs11060180	122819039	G	OGFOD2/CCDC62
14	rs11158026	54882151	T	GCH1
14	rs8005172	88006268	T	GALC/GPR65
15	rs2414739	61701935	G	VPS13C
16	rs14235	31110472	A	ZNF646/KAT8/BCKDK
16	rs4784227	52565276	T	TOX3/CASC16
18	rs12456492	43093415	G	SYT4/RIT2
20	rs55785911	3172857	A	DDRGK1
22	rs174674	19946502	A	COMT
22	rs740603	19957654	A	COMT
22	rs165599	19969258	G	COMT

### Selected imaging and genetic features from LASSO

The number of features selected for each training fold was not constant, ranging from 7 to 20. In some cases, no genetic features could be selected. For imaging features, we identified FA of 3 ROIs related to the AAO of PD for all five folds. The three selected regions were as follows: right inferior frontal gyrus opercular part (IFGoperc), left caudate nucleus (CAU), and left PAL. For genetic features, the selected SNP was only rs4653767 (CHR: 1; BP: 226728377; MA:C; associated gene: ITPKB), which was found more than three times in five folds.

### Comparison of prediction models using the three approaches

**Figure 3:**
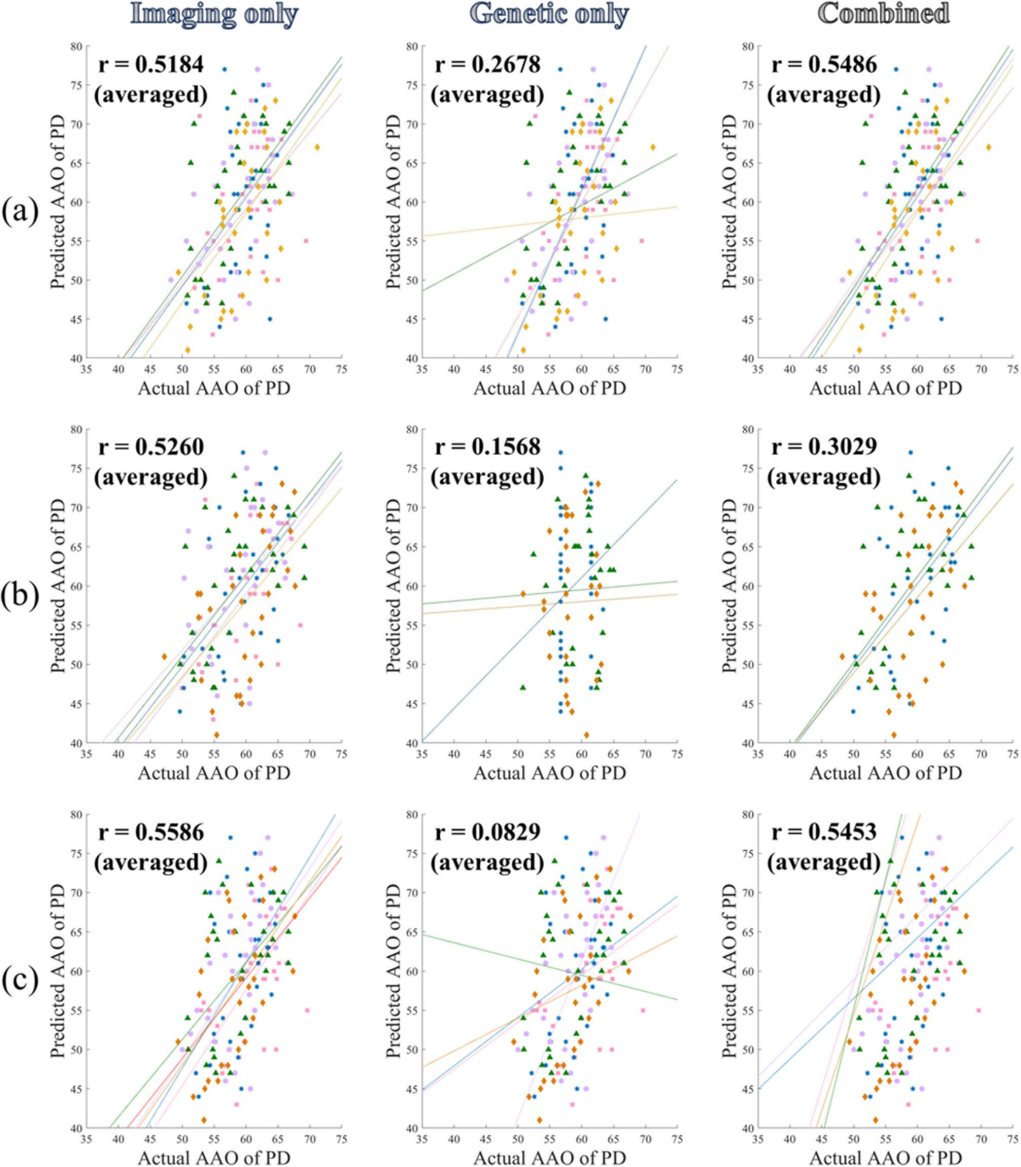
Prediction plots using different approaches. The actual and predicted AAO of PD using various approaches. Our models were compared with those using mRMR and LASSO. (**a**) The prediction plot using our os-SCCA; (**b**) the prediction plot using mRMR; (**c**) the prediction plot using LASSO. There were five colored lines and dots. Each represents a different left-out fold. The solid lines represent a linear fit of the corresponding data.

We constructed random forest regression models using the selected imaging, genetics feature, and the combined features from os-SCCA to explain the AAO of PD in a five-fold cross-validation. Additional prediction models were built using the features obtained from mRMR and LASSO approaches and compared with those of os-SCCA. Using imaging features only, all three approaches worked well. Our proposed model showed a meaningful correlation (r = 0.5184, *p* = 0.0105; averaged) between the predicted and real AAO of PD over five left out test folds. The mean RMSE between the predicted and real AAO of PD was 8.1197. Using mRMR resulted in a correlation of r = 0.5260 with *p* = 0.0026, while using LASSO led to a correlation of r = 0.5586 with *p* = 0.0022. The prediction plots are displayed in the first column of Fig. 3. The correlation values derived from mRMR and LASSO were higher than our approach partially. A potential reason behind this trend is given in “Discussion” section. The mean RMSE values were 8.1652 using mRMR and 7.9354 using LASSO.

Using genetic features only, the models were less successful than using imaging features (Fig. 3b). The LASSO failed to identify genetic features in some cases, and there were only a few features that had high enough FS scores. Our proposed model showed a modest correlation (r = 0.2678, *p* = 0.1364; averaged) between the predicted and real AAO of PD over five left out test folds. The mean RMSE between the predicted and real AAO of PD was 9.1678. Using mRMR resulted in a correlation of r = 0.1568 with *p* = 0.4457, while using LASSO led to a correlation of r = 0.0829 with *p* = 0.6684. In the case of LASSO, there were even negative values of r. The correlation values derived from our approach were higher than those derived from mRMR and LASSO. The second column of Fig. 3 displays these results. The mean RMSE values were 9.1678, 9.4043, and 10.1420 when using os-SCCA, mRMR, and LASSO, respectively.

Using both imaging and genetic features, our model showed prediction improvement (increase in correlation between real and predicted values) compared to using only imaging features. However, using mRMR and LASSO led to degraded performance (decrease in correlation) compared to using only imaging features even if more features were adopted. Our proposed model showed an improved correlation (r = 0.5486, *p* = 0,051; averaged) between the predicted and real AAO of PD over five left out test folds. The mean RMSE between the predicted and real AAO of PD was 7.9764. Using mRMR resulted in a correlation of r = 0.3029 with *p* = 2,186, while using LASSO led to a correlation of r = 0.5453 with *p* = 0.0025. The prediction plots using both imaging and genetic features are shown in the third column of Fig. 3. The mean RMSE was 11.8779 (mRMR), 8.0273 (LASSO), and 7.9764 (our approach). Overall, we found that our approach identified more meaningful features by fully exploring the three pair-wise associations present in the data.

## Discussion

To the best of our knowledge, this is the first study to report on the predictions of AAO of PD in the field of imaging genetics. Recent clinical trials in PD have focused on neuroprotective treatment. Therefore, the early diagnosis and prediction of PD are becoming more important^24^. Various non-motor symptoms could present even before parkinsonian motor symptoms and be regarded as markers for PD development^25,26^. However, these non-motor symptoms are not PD-specific symptoms, and common in both PD patients and normal elderly subjects^1^. Many previous studies also tried to investigate protein biomarkers in the cerebrospinal fluid of PD patients^27,28^, but these proteins are only available from an invasive procedure with possible side effects. Further, these studies analyzed protein biomarkers in PD patients after diagnosis. Therefore, all these results were based on the neurodegenerative changes of PD. Similarly, although neuroimaging techniques are non-invasive, the related structural changes are the results of neurodegeneration in PD. Therefore, those neuroimaging and protein biomarkers might not reflect the neurodegenerative changes associated with disease risk but could be the result of neurodegenerative changes^29^. By using neuroimaging and SNP data simultaneously, our prediction model might reflect not only the results of neurodegeneration but also the neurodegenerative changes associated with disease risk. This is because SNP data are available from birth. Two types of data in our study, genetic and neuroimaging information, are becoming widely available in healthcare, meaning that our model could be useful in clinical practice.

In this study, we adopted a multivariate model of os-SCCA to capture the associations of the imaging–genetic, genetic–target, and imaging–target pairs. This approach considers imaging and genetic data simultaneously to capture the multivariate nature of the data better, reduces the number of statistical tests, and regularizes high dimensional data. The resulting features were used to predict the AAO of PD, and the performance was enhanced if the features derived from os-SCCA were used. If each of the resulting brain regions, genes, and the interactions of brain region and gene from our study are studied in more detail, these resulting features could be used as biomarkers related to the prodromal period of PD. Also, our study requires fewer samples than conventional GWAS as there are not a lot of univariate tests. Future studies using more samples from independent cohorts are necessary to validate our findings fully.

The prediction models that used only imaging features showed a high correlation for all three approaches. The correlation values derived from mRMR and LASSO were higher than our approach partially due to the nature of the mRMR and LASSO. This may be because LASSO and mRMR are tailored for extracting continuous features (e.g., imaging features) directly related to AAO as the outcome. This translates to optimizing only the imaging–target associations. However, os-SCCA optimizes additional genetic–target and imaging–genetic associations simultaneously, which leads to holistic modeling of the multimodal data. The strengths of os-SCCA were well demonstrated in the predictive model with genetic features. Therefore, it becomes challenging to select a few related to the outcome. Using LASSO, there were folds with no genetic features selected. Using mRMR, although we ensured that the same number of features were used as in os-SCCA, the FS of the selected genetic features were very low, which might obscure the selection process. Only with jointly optimizing the genetic–target and imaging–genetic associations using our approach, could meaningful genetic features be selected. We were able to predict AAO with an RMSE of less than 10 years using our approach. This implies that approximate estimation at a 10-year interval is possible. Our results show that it was possible to predict the AAO of PD using only the genetic features from os-SCCA. These findings could be used as potential biomarkers related to AAO of PD, and help elucidate the associated pathomechanism of PD. Still, the identified SNPs need to be confirmed in additional validation studies with more samples. Most patients in our study have AAO between 50 and 60. Thus, we constructed a simple additional model using only the sample mean of all data (i.e., mean of AAO = 61.35). The RMSE was 9.4689, which fared worse than those using both imaging and genetic features (7.9764), only imaging features (8.1197), and only genetic features (9.1678).

The effectiveness of os-SCCA was seen by evaluating the performance of the predictive model using both genetic and neuroimaging data. By considering all thee pair-wise associations in selecting the features, the performance improved over models that used only imaging and genetic features in every fold. In models using features from mRMR, even if the model could use more features than those of os-SCCA, the performance decreased. In the case of LASSO, the performance was lower than os-SCCA. Overall, we confirmed that imaging and genetic features should be selected, considering all possible associations when using genetic and neuroimaging data at the same time in an imaging genetic framework.

We identified 25 genes annotated from 24 SNPs and 14 brain regions in all training sets. One of the resulting genes, NUCKS1, is known to have a functional association in the brain of people with PD. A significant association of expression and transcription levels of NUCKS1 with PD has been observed^30,31^. ACMSD is associated with aging and risk of PD, tentatively suggesting that this enzyme might influence pathogenesis^32^. Additionally, COMT, an enzyme involved in the degradation of dopamine, is a critical determinant of the availability of this neurotransmitter in the prefrontal cortex. COMT modulates the activity of the enzyme, affects cognition, and is magnified in the aging brain^33^. There are also studies showing that COMT is related to the late-onset of PD, and COMT is a modifier of the AAO in PD^34,35^. We investigated rationales for the identified brain regions. The PCG and PreCG are the regions that have age-related differences in the structure of the brain^36,37^. Other brain structures such as THA found in our study were also reported in early PD patients^38,39^. The ratio of dopamine transporter binding of PUT is reported to be related to the difference between old-onset PD and young-onset PD groups^40^. In particular, PCG is one of the regions that showed substantially different gene profile changes with age^41^. As symptoms of PD vary according to AAO, an in-depth study of the brain regions of our study combined with the PD symptoms might contribute to the enhanced characterization of PD subtypes and possibly lead to a redefinition of the subtypes^42^. Considering that neurodegenerative changes start a few decades before AAO, diverse changes in various brain regions are quite feasible. However, most previous studies have focused on the brain structure related to clinical features in PD patients, and very few of them assessed the association between AAO and brain structure in PD patients. Clinical characteristics of young-onset PD are already well-known from many previous studies^2–4^, but it was difficult to determine whether the relevant brain structures in our study were from AAO itself or were also linked with clinical features in young-onset PD patients. It is challenging to control for all the clinical diversity present in PD patients, and our results were consistent with previous studies which revealed the known involved structure in early PD patients. Considering there is no previous study focused on the AAO of PD, our results should be interpreted cautiously. Our primary objective was related to AAO of PD, and we did not distinguish between healthy controls (HC) and PD. This was partly because there was an ambiguity in HC from the PPMI database. The PPMI data are not follow-up data with a long duration, thus HC cases could develop PD in the future or stay healthy. This can be examined further in future studies using well-designed follow-up data. A longitudinal study is better suited for AAO of PD as it might offer more accurate AAO information of PD along with neuroimaging data near the time when neurodegeneration starts.

Our study has some limitations. Our results were obtained using a cross-sectional design. Therefore, it is difficult to distinguish between PD progression and risk. Although imaging genetic analysis needs a smaller sample size compared to GWAS, we recruited a relatively small number of patients. Even with these limitations in study design and sample size, we were able to suggest significant prediction models for AAO of PD using genetic and neuroimaging features from os-SCCA. We hope this study is the first step to identifying the pathomechanism and related genetic factors in PD patients. Second, PD is a wide-spectrum disorder with diverse motor and non-motor symptoms^1^. Considering that AAO is related to various parkinsonian symptoms and progression, it is difficult to eliminate all the confounding factors. Therefore, it is difficult to control for all possible clinical characteristics and to focus only on those relevant to AAO. In a similar vein, we identified brain imaging–SNP pairs related to AAO in PD using only SNPs related to PD to make feature selection and interpretation tractable. To use the os-SCCA for other purposes, a similar reduction in feature dimension of the input modalities might be necessary especially for the genetic factors. Lastly, no replication using another dataset is a limitation of our study. Our approach requires multimodal data requirements and we could not find any open database satisfying our data requirements to the best of our knowledge.

In this study, we investigated relevant genetic factors and brain regions associated with the AAO of PD and suggested a prediction model using os-SCCA. Our study jointly models genetic factors and neuroimaging and thus could be a more suitable model compared to studies using genetic factors and neuroimaging only. Our proposed os-SCCA is a special case of TSCCA. Thus, the scalability of TSCCA applies. Our approach could be extended to included additional imaging modalities hence becoming four-way SCCA. TSCCA belongs to the family of linear models and thus could be easily scaled in terms of computational speed and size of data sets. We hope our study can be the first step toward accurate early diagnosis and improved therapy for PD.

## Data Availability

The imaging and phenotypic data are available from the PPMI database (http://www.ppmi-info.org). Interested researchers should contact the database administrator to request access to the data.

## Code availability

The os-SCCA code is available through the repository at https://github.com/Ji-Hye-Won/os-tscca.
